# A Case of Disseminated Histoplasmosis Presenting in a 65-Year-Old Male Without Apparent Immunodeficiency Successfully Treated With Isavuconazole

**DOI:** 10.7759/cureus.53495

**Published:** 2024-02-03

**Authors:** Colton P Boney, Ali Hassoun, Sandhya Viswanathan, Rabi Shrestha

**Affiliations:** 1 Infectious Disease, Alabama College of Osteopathic Medicine, Huntsville, USA; 2 Infectious Disease, Alabama Infectious Disease Center, Huntsville, USA; 3 Infectious Disease, Sri Ramachandra Institute of Higher Education and Research, Chennai, IND; 4 Internal Medicine, Crestwood Medical Center, Huntsville, USA

**Keywords:** histoplasmosis, isavuconazole, clinical mycology, oral histoplasmosis, adrenal histoplasmosis

## Abstract

*Histoplasma capsulatum* causes symptoms in fewer than 5% of infected people, with most recovering without treatment two to three weeks after the onset of symptoms. Progressive disseminated histoplasmosis in adults occurs most often in persons with underlying immunodeficiency.

We present a case of a 65-year-old caucasian male without any known immune defect from North Alabama, United States, presenting with chronic tongue ulcer and constitutional symptoms. CT and positron emission tomography scans showed disseminated infection with pulmonary nodule, oral/buccal lesions, and bilateral adrenal hyperplasia. The patient's left adrenal gland and tongue were biopsied and stains confirmed the presence of histoplasmosis in both samples.

The patient was treated with isavuconazole off-label as per the United States FDA. The patient tolerated the therapy well and had symptomatic improvement. A follow-up CT scan showed improvement and resolution of adrenal masses.

## Introduction

*Histoplasma capsulatum* is the most common systemic fungal infection in the United States. The dimorphic fungus is typically associated with immunosuppression or significant environment source exposure [1]. Infections are common, but typically are asymptomatic and resolve without treatment. Furthermore, extrapulmonary dissemination is even more rare for patients without underlying immunological disease. This report details a case of disseminated histoplasmosis in an individual living in an endemic area. Initial laboratory testing and history failed to identify any immune defect or predisposing chronic conditions in this patient. Additionally, this patient’s serology for histoplasmosis was negative. It is not uncommon for patients with histoplasmosis to test negative on serology due to the varying sensitivity for immunologic/antigen markers of histoplasmosis [2,3]. However, the lack of discernible risk may have otherwise delayed care for this patient’s infection. The case emphasizes a need for a high index of suspicion even in the absence of traditional risk factors or false negative serology.

Isavuconazole was implemented off-label to treat the patient. It was well-tolerated leading to the resolution of symptoms and a reduction in the adrenal masses. Isavuconazole was chosen over itraconazole, given its favorable in-vitro evidence and increased tolerability [4-7]. 

In short, this case report contributes to the understanding of disseminated histoplasmosis, comprehensive diagnostic evaluation, and the exploration of alternative antifungal therapies for presentations in immunocompetent individuals.

## Case presentation

A 65-year-old white male presented to his dentist for soreness of his left tongue for several weeks with a newer onset of difficulty swallowing. He had a past medical history of hypertension that was well-controlled on lisinopril. He had a 30-pack-a-year history of smoking and actively smoked half a pack per day, but had no diagnosed pulmonary disorders and denied cough or shortness of breath. He had never traveled outside of North Alabama where he worked in construction. He had a family history on his maternal side of hypertension and coronary artery disease.

**Figure 1:**
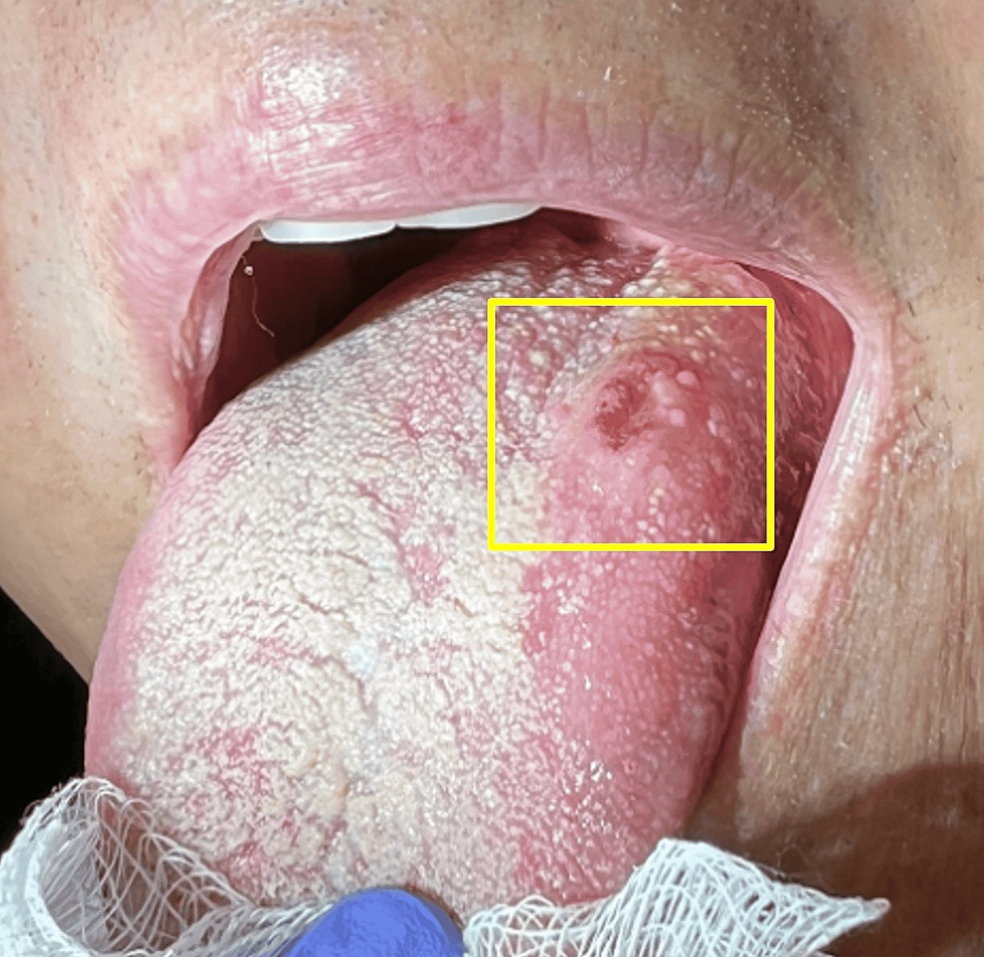
Tongue lesion (within the yellow box) prior to biopsy. This ulcerated lesion failed to resolve with the trial of oral nystatin solution by dentistry.

His dentist noticed a lesion that was originally believed to be an aphthous ulcer. As the pain continued following that evaluation, the patient was put on a course of nystatin. After five weeks without symptom resolution, the patient was referred to ENT for a biopsy of the tongue lesion as pictured in Figure 1. The biopsy showed background benign pseudoepitheliomatous hyperplasia but was negative for p40 malignant cells that would indicate squamous cell carcinoma. Pathology did not note other malignant features of the tongue biopsy. Stains were negative for mycobacterial diseases. Grocott's methenamine silver stain (GMS) and the periodic acid-Schiff stain (PAS) revealed yeast consistent with histoplasmosis, at which point, he was referred to infectious disease.

Upon infectious disease evaluation, the patient had been experiencing constitutional symptoms such as intermittent fever, weight loss, fatigue, and night sweats. The patient also noted he had episodes of profuse sweating and found it odd that the episodes had no correlation to heat exposure. In his estimation, these symptoms had been ongoing for the past seven weeks. A review of systems failed to identify any additional symptoms, and the physical exam was unremarkable.

Given the biopsy results, the patient was immediately started on isavuconazole 372 mg once a day by mouth. Baseline labs were drawn to check for Histoplasma antibodies/antigens, immune competency, and HIV status. The results of his relevant labs are documented in Table 1. A chest CT scan was also ordered to check for pulmonary or disseminated infection.

**Table 1:** Lab work This patient notably did not have leukocytosis or reactive antigen testing for histoplasmosis. Mild deviance from the reference range was noted with slightly elevated CD4 count and IgM levels, and mild anemia. Despite the lack of typical findings for disseminated histoplasmosis, the patient's elevated sed rate, CRP, and alkaline phosphatase were highly indicative of systemic disease.

Test	Patient's result	Reference range
WBC count	5.51	4.10-12.20 x10*3/uL
Platelet count	130	137-352 x10*3/uL
RBC count	4.14	4.40 – 5.50 x10*6/uL
CD4 count	67	30-61 cells/mm*3
Erythrocyte sedimentation rate (sed rate)	65	0-20 mm/h
C-reactive protein (CRP)	2.0	<0.5 mg/dL
Alkaline phosphatase	210	39-117 U/L
Histoplasma antibody, serum	Negative	Negative
Histoplasma antigen, urine	Negative	Negative
IgM	235	40-230 mg/dL
HIV antigen/antibody	Negative	Negative

**Figure 2:**
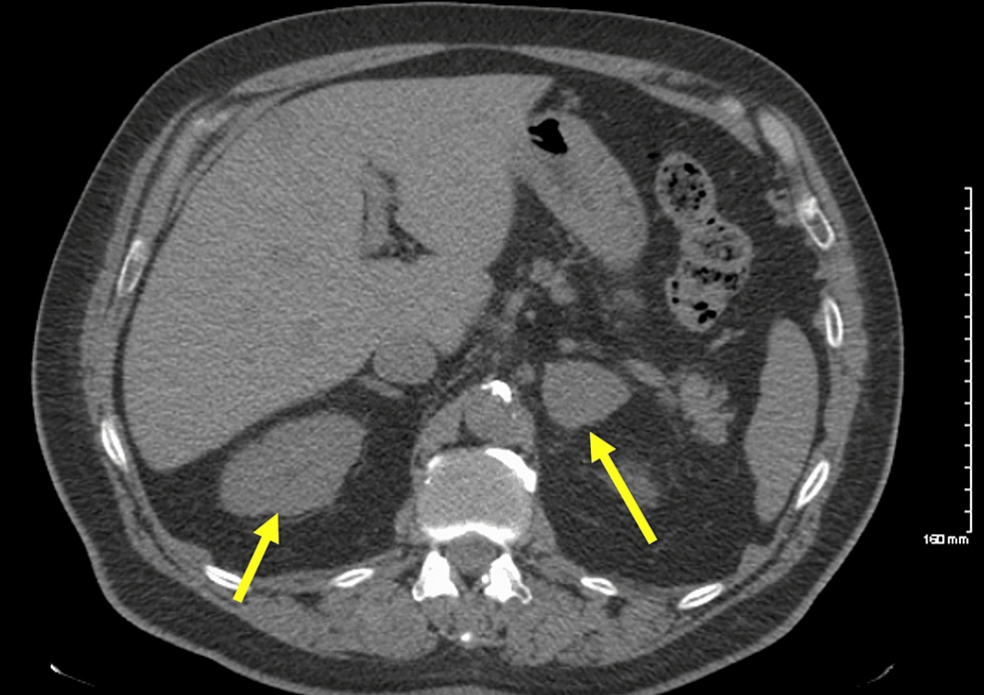
Abdominal CT with yellow arrows indicating each adrenal gland. This abdominal CT scan shows bilateral adrenal hyperplasia as per the radiology report. This scan was performed prior to the CT-guided biopsy of the left adrenal gland or the subsequent treatment with isavuconazole.

Lab work showed negative for HIV, normal IgG levels, and negative serum antigen/antibody for histoplasma. Complete blood count and metabolic panel were fairly unremarkable and showed few abnormalities. Abnormalities noted were an alkaline phosphate of 209 U/L, AST of 40 U/L, and a mild thrombocytopenia at 130 per uL. The patient's CD4 count was mildly elevated. Inflammatory markers were also elevated with a C-reactive protein (CRP) of 2.0 mg/dL and an erythrocyte sedimentation rate (sed rate) of 65mm/hr. A chest CT without contrast was compared to a CT done six years prior and revealed a new noncalcified nodule of the medial basal segment of the right lower lobe measuring 1.8 x 1.1 cm. There were also new bilateral adrenal masses which raised concerns for disseminated infection or malignancy (Figure 2). A positron emission tomography (PET) scan was ordered to evaluate the nature of the masses seen on CT. Increased fluorodeoxyglucose (FDG) uptake was noted at the left tongue and jawline. Both adrenal masses were significantly hypermetabolic with a standardized uptake value (SUV) of 11.1. The pulmonary nodule noted on the CT scan did not show abnormal or asymmetric activity. These findings led to a CT-guided biopsy of the left adrenal gland that showed significant histoplasmosis per pathology.

Isavuconazole 372mg once daily was continued for one year. During this time, the patient was followed monthly for recurrence of symptoms and toleration of medication. Repeated lab work showed little variance from the original evaluation and no increases in liver function tests as a result of antifungal medication. CT scans were repeated at three and 10 months of therapy duration. Complete resolution of the right adrenal mass was noted at three months of therapy. The left adrenal mass decreased from an initial measurement of 5.2 x 4.8 cm to 3.9 x 3.6 cm at 10 months of therapy. CT scans detected no change in morphology or size of the pulmonary nodule. The majority of the patient’s symptoms improved; however, he still complained of inappropriate sweating which had only slightly improved over the course of treatment. Outpatient evaluation for adrenal insufficiency is currently underway as the urine metanephrine test was within normal limits.

## Discussion

Histoplasmosis continues to be the most common systemic fungal infection in the United States. The dimorphic fungus is an endemic organism and is seen most commonly in the Ohio and Mississippi River valleys of the United States [8]. The fungus is found in organic material, such as the excrement of bats and birds. Thus, it is often found in decaying organic material. Inhalation of spores is the most common route of exposure. Histoplasmosis is commonly seen in those with underlying immune suppression, such as those undergoing cancer treatment, immunomodulatory drug use for autoimmune diseases, and those with untreated HIV infection [9]. While not exclusive to those with immune suppression, the presence of histoplasmosis should cause concern for other immunological diseases. In this patient’s case, no such immunomodulating disease or immunosuppressive therapy was present. Once inside the body, histoplasma can spread to multiple organ systems, causing a disseminated infection. These disseminated infections can become life-threatening and disproportionately affect those with severe immunocompromising status or children less than two years of age [1,8].

The patients diagnosed with histoplasmosis often have some attributable exposure to the pathogen including hiking or spelunking in endemic areas, close contact with bats or birds, farming or gardening, etc. For this patient, his work in construction is a well-documented risk factor for histoplasmosis [8]. Likewise, smoking is a known risk factor for developing pulmonary histoplasmosis even in the absence of chronic obstructive pulmonary disease (COPD) diagnosis [10,11]. However, this patient had an otherwise healthy immune system. For most patients, immunocompetence can often prevent the common acute pulmonary histoplasmosis from disseminating to other organ systems [12,13]. Initially, the evaluating clinicians were rightfully concerned about malignancy leading to his CT and PET scan. It is important to recognize the role of pathology in the diagnosis of this patient’s histoplasmosis. For a patient with no immunological dysfunction and negative antigen/antibody testing for histoplasmosis, correctly identified biopsies provided his diagnosis where it may have otherwise not been considered. There is some existing evidence that shows the sensitivity and specificity of biopsies for histoplasmosis varies with the experience of the pathologist reviewing the biopsy [2,3,14]. Those seeing more cases in endemic regions may be less likely to miss the diagnosis as their familiarity with the disease presentation increases. Additionally, fungal cultures often take several weeks to show meaningful and interpretable growth for identification of the fungal pathogen. This delay in result per culture can often delay treatment and allow further progression of the disease. Given this and the Histoplasma found in both biopsies performed to exclude malignancy, fungal cultures were not ordered.

Isavuconazole was tolerated well and led to symptomatic improvement for this patient. This would be considered off-label for the treatment of histoplasmosis as the drug is currently only FDA-approved for aspergillosis and mucormycosis. More data would be needed to evaluate isavuconazole's efficacy in histoplasmosis. In the VITamin D and OmegA-3 TriaL (VITAL) of 2016, the drug showed efficacy against multiple dimorphic fungal infections and several case reports exist showing its efficacy in isolated histoplasmosis granulomas or as salvage therapy for those who have failed amphotericin B and/or itraconazole [15-17]. Itraconazole is also known to have side effects decreasing tolerability and causing adverse events in up to 35% of patients [18]. In these instances, providers often have to weigh the adverse event risks of continuing itraconazole or changing to fluconazole despite poor susceptibility. Studies have also shown isavuconazole has a preferable minimal inhibitory concentration (MIC) to histoplasmosis as compared to fluconazole [7]. Given the increased tolerability of the drug and in-vitro evidence for histoplasmosis sensitivity, isavuconazole was chosen over itraconazole or fluconazole for this patient. As the patient had no central nervous system involvement seen in the PET scan or neurological defect suggestive of spread into the central nervous system, there was no evidence that the patient needed to be escalated to treatment using amphotericin B. At one year of treatment, the patient shows no signs of drug toxicity or signs of antimicrobial failure.

## Conclusions

This case reiterates the clinical importance of biopsy in those with suspected malignancy or fungal infection. In the absence of positive serological testing or known immune defect, this patient’s disseminated histoplasmosis may not have been suspected till further organ involvement occurred. Additionally, this patient’s improvement with the use of isavuconazole provides some evidence that further studies should be done with other antifungal agents for histoplasmosis given limited treatment options.
